# Comparative Analysis of Immune Gene Transcription in Sea Bream (*Sparus aurata*) Challenged with RGNNV or RGNNV/SJNNV Betanodaviruses

**DOI:** 10.3390/pathogens13060478

**Published:** 2024-06-04

**Authors:** Juan Gemez-Mata, Patricia Moreno, Daniel Alvarez-Torres, Esther Garcia-Rosado, Julia Bejar, M. Carmen Alonso

**Affiliations:** 1 Instituto de Biotecnología y Desarrollo Azul (IBYDA), Universidad de Málaga, 29071 Málaga, Spain; 2 Departamento de Microbiología, Facultad de Ciencias, Universidad de Málaga, 29071 Málaga, Spain; 3 Departamento de Biología Celular, Genética y Fisiología, Facultad de Ciencias, Universidad de Málaga, 29071 Málaga, Spain

**Keywords:** European sea bream, RGNNV, RGNNV/SJNNV, innate immune response

## Abstract

Gilthead sea bream and European sea bass display different resistance–susceptibility patterns during infection with different nervous necrosis virus (NNV) species, which may derive from differences in the triggered immune response. Based on this premise, we analysed the transcription of several selected immune-related genes in sea bream experimentally infected with NNV isolates obtained from sea bass (DlNNV, RGNNV) or sea bream (SaNNV, RGNNV/SJNNV). Viral replication only occurred in SaNNV-inoculated fish; therefore, the differences between the immune response elicited by both viruses may be the key to understanding the mechanism behind the inhibition of DlNNV replication. Principal component analysis clustered samples according to the viral isolate from 1 day post infection onwards and evidenced differences in the immune response against both viruses, even though no mortalities or symptoms were recorded. The response against DlNNV is characterized by higher *rtp3* transcription early after the infection, longer-lasting *il-10* transcription and stronger induction of *casp1* and *hsp70*. These genes should be targets for future studies in order to elucidate their role in hampering NNV replication in sea bream, which is essential for developing effective prophylactic measures.

## 1. Introduction

Viral nervous necrosis (VNN) is one of the main threats for the Mediterranean fish farming industry [1]. Affected fish display vacuolation and necrosis in cells in brain, spinal cord and retina [2]. These lesions are responsible for alterations in swimming behaviour and, eventually, fish death. The causative agent of this disease is the nervous necrosis virus (NNV), a naked, positive-sense, single-stranded RNA virus belonging to the *Nodaviridae* family and *Betanodavirus* genus. The genome of this virus is composed of two segments: RNA1, which encodes the 100-kDa RNA-dependent RNA polymerase (RdRp); and RNA2, encoding the 42-kDa capsid protein (CP). In addition, a sub-genomic segment, RNA3, is synthesized during RNA replication from the RNA1 3′-end. RNA3 comprises two open reading frames that encode two non-structural proteins, B1 and B2. Based on the partial sequence of the *cp* gene, betanodaviruses have been clustered into four species (former genotypes) [3]: red-spotted grouper (RGNNV), striped jack (SJNNV), barfin flounder (BFNNV) and tiger puffer (TPNNV) nervous necrosis virus. In addition, reassortment between RGNNV and SJNNV segments has also been reported. Thus, SJNNV/RGNNV or RGNNV/SJNNV (RNA1/RNA2) betanodaviruses have been isolated from or detected in European sea bass (*Dicentrarchus labrax*), Mediterranean horse mackerel (*Trachurus mediterraneus*), common sole (*Solea solea*), Senegalese sole (*S. senegalensis*), hybrid groupers (*Epinephelus fuscoguttatus* and *E. lanceolatus*) and gilthead sea bream (*Sparus aurata*) [4,5,6,7,8,9,10,11,12,13].

Although NNV can affect a wide range of fish species worldwide, not all viral species are equally virulent to all susceptible fish species, and this issue is especially relevant concerning European sea bass and gilthead sea bream, which are often cultured in close proximity to each other in Mediterranean aquaculture facilities. Thus, sea bass has been reported to be highly susceptible to RGNNV [14,15,16], whereas RGNNV/SJNNV isolates cause very low mortality in this fish species [15,16]. On the contrary, RGNNV/SJNNV reassortants have been isolated from outbreaks reported in cultured sea bream, causing high mortality at the larval stage [10,11,13,17,18], whereas this fish species does not suffer disease after RGNNV infection. In fact, RGNNV/SJNNV reassortants seem to have special adaptation to sea bream, mainly at the larval stage, and, therefore, they may constitute a new threat for the Mediterranean aquaculture.

This difference in the severity of the disease caused by different betanodaviruses in the same susceptible host underlines discrepancies concerning virus–host interaction. Therefore, the analysis of immune gene transcription after infection with different betanodaviruses in the same fish species can be crucial to identify genes with a pivotal role in the control of the infection. In addition, those genes can be the key to developing effective prophylactic measures. 

The present work analyses the transcription of selected immune gene in sea bream experimentally infected with RGNNV (isolated from sea bass, DlNNV) or RGNNV/SJNNV (isolated from sea bream, SaNNV) isolates. The genes analysed have been those suggested to be relevant in controlling RGNNV infection in sea bass: *irf3*, *rtp3*, *sacs*, *il-1β*, *il-6*, *il-10*, *tnf-α*, *granb*, *casp1* and *hsp70*.

## 2. Material and Methods

### 2.1. Viral Propagation and Titration

The viral isolates SpDl-IAusc965.09 (hereafter DlNNV, RGNNV, obtained from diseased sea bass) and SaNNV (RGNNV/SJNNV, isolated from diseased sea bream; RNA1 GenBank accession no. PP460508; RNA2 GenBank accession no. PP460509) were used in this study. Both viruses were propagated on E11 cells [19] in Leibovitz L-15 medium (Gibco, Paisley, UK) supplemented with 2% foetal bovine serum (FBS, Gibco, Paisley, UK), 100 units/mL penicillin and 10 mg/mL streptomycin (Sigma, Steinheim, Germany). Inoculated cells were incubated at 25 °C until extensive cytopathic effects (CPEs) were observed. The resulting viral suspensions were titrated on E11 cells at 25 °C according to the 50% tissue culture infective dose (TCID_50_) method [20] and subsequently diluted in L-15 medium to inoculate sea bream specimens.

### 2.2. Fish Trial

Fish used in this challenge were proven to be RGNNV and SJNNV free according to Lopez-Jimena et al. [21]. Sea bream specimens (3 g, average weight) were allocated in the following groups in duplicate (each replica was used for sampling or mortality recording, n = 30): (i) L-15-injected fish (control group), (ii) DlNNV-challenged fish and (iii) SaNNV-challenged fish. Both viruses were inoculated at 2.5 × 10^5^ TCID_50_/fish by intramuscular injection. Fish were maintained under the following conditions: 24 ± 0.5 °C, pH 7.5 ± 0.5, salinity 38 ± 1 ppt, oxygen 6 ± 1 mg/L, NO_2_ < 3 mg/L and light 12 h. Fish were fed three times a day, and the food supply was adjusted to a maintenance ration (1–2% of the biomass).

For fish sampling, six animals per group were euthanized by anaesthetic (MS-222, Sigma, Steinheim, Germany) overdose at 1, 3 and 7 days post-inoculation (dpi). Fish for mortality recording were maintained under the above-described conditions for 30 days. Brains from sampled and survivor (n = 6) fish were aseptically collected and individually stored in TRI reagent (Sigma-Aldrich, Steinheim, Germany) at −80 °C until used.

### 2.3. Sample Processing

Brains were separately homogenized in TRI reagent using an MM400 homogenizer (Retsch, Haan, Germany). Total RNA was obtained following commercial guidelines and quantified using DS-11FX (DeNovix, Wilmington, DE, USA). RNA quality was measured by the absorbance ratios A260/230, between 2.0 and 2.4, and A260/280, between 1.8 and 2.1. Finally, RNA was treated with RNase-free DNase I (Sigma, Steinheim, Germany) and reverse-transcribed with the Transcriptor First Strand cDNA Synthesis Kit (Roche, Mannheim, Germany), as specified by manufacturer’s instructions. Resulting cDNA was stored at −20 °C until used.

### 2.4. Quantification of the RNA1 Viral Segment

Virus replication in brain was analysed by absolute real-time PCR using the primers shown in Table 1, which amplify a fragment within the *RdRp* gene (RNA1 segment). Amplifications were carried out according to Lopez-Jimena et al. [22], using a LightCycler 96 Thermocycler (Roche, Mannheim, Germany) and the Fast Start Essential DNA Green Master Mix (Roche, Mannheim, Germany), in 20-µL mixtures (final volume) containing cDNA generated from 50 ng of RNA. Amplification profile consisted of 95 °C for 10 min, followed by 45 cycles at 95 °C for 10 s, 52 °C for 10 s and 72 °C for 10 s. Melting curves were obtained at 95 °C for 10 s, 65 °C for 60 s and 97 °C for 1 s. Serial dilutions of the pJET vector containing the complete NNV RNA1 sequence were used as reference standard curve.

### 2.5. Quantification of Immune Gene Transcription

The transcription of the following genes was analysed: interferon regulatory factor 3 (*irf3*), receptor transporter protein 3 (*rtp3*), sacsin (*sacs*), interleukin 1 beta (*il-1β*), interleukin 6 (*il-6*), interleukin 10 (*il-10*), tumour necrosis factor alpha (*tnf-α*), granzyme B (*granb*), caspase 1 (*casp1*) and heat-shock protein 70 (*hsp70*). Transcription was quantified by qPCR using the LightCycler 96 Thermocycler and 20-μL mixtures containing 10 µL of the Fast Start Essential DNA Green Master Mix, 1 μL of each primer (0.75 μM, final concentration) (Table 1) and 2 µL of cDNA (50 ng). The amplification profile was as follows: 95 °C for 10 min, followed by 40 cycles of 65 °C for 10 s, 60 °C for 10 s, 72 °C for 10 s and a final step of 95 °C for 10 s, 65 °C for 60 s and 97 °C for 1 s. Fold change values (FC) were calculated by the 2^−∆∆Ct^ method, using beta actin (*actb*) as the endogenous gene (Table 1). The reference sample for FC calculation was a brain sample collected at 1 dpi from L-15-injected sea bream.

**Table 1:** Primers used in this study.

Gene	Primer	Sequence (5′-3′)	Amplicon Size (bp)	Reference
*RdRp*	F	GGCTCAGATCTGGTAATGTTTCAA	63	[22]
	R	CAAAGCCAAGGGAAGAAGCA		
*irf3*	F	TCAGAATGCCCCAAGAGATT	107	[23]
	R	AGAGTCTCCGCCTTCAGATG		
*rtp3*	F	CAGGTGCAGCAAGTGTGGGA	120	This study
	R	GTCTCACCTTGACCACGCCC		
*sacs*	F	ACATCCGGACCTTGGTGCCT	124	This study
	R	AGCGGTGGTGTAGTCTGTCCA		
*il-1β*	F	AGCGCAGTAGAAGAGCGAAC	117	[24]
	R	CACTCGGACTAAGTGCCTCTG		
*il-6*	F	CCAGATCCCCTCAAGATTCA	144	[24]
	R	AAGGTGTCGGAGCTGTCG		
*il-10*	F	CAGGCCATGAACAACATCC	143	[24]
	R	TGGACGTCAGATTTGAGCTG		
*tnf-α*	F	TTCCGACTGGTGGACAATAAG	143	[24]
	R	GAGATCCTGTGGCTGAGAGG		
*hsp70*	F	TGAGGTAAAGTCCACTGCCGGA	132	This study
	R	AGCTCTCTTGTTGTCGCTGATGT		
*granb*	F	GAAACAAAGGAACGGGTCAA	128	This study
	R	GAGCTGTCCATCTTTTGCTTG		
*casp1*	F	TCGAAGAGACGGACAGTGTG	123	[24]
	R	CGTTGATGGGGAACTCATCT		
*actb*	F	ATTGTCAAACTGCACCCACA	139	[24]
	R	GCTCAACAGCCTTGATGACA		

### 2.6. Statistical Analyses

Results were statistically examined using the GraphPad Prism 6.01 software (GraphPad Software, Inc., La Jolla, CA, USA). Viral genome quantification was analysed using the one-way ANOVA test and Bonferroni’s multiple comparison test as a post-test. Immune gene transcription was analysed using the two-way ANOVA test. Normal distribution of data was verified by the Shapiro–Wilk test. Tukey’s multiple comparison analysis was carried out as a post hoc test. Principal component analysis (PCA) was applied to correlate variables determined in the brain of infected fish using SPSS v.28 software. Values of *p* < 0.05 were considered significant.

## 3. Results

### 3.1. Fish Mortality and Viral Replication

**Figure 1:**
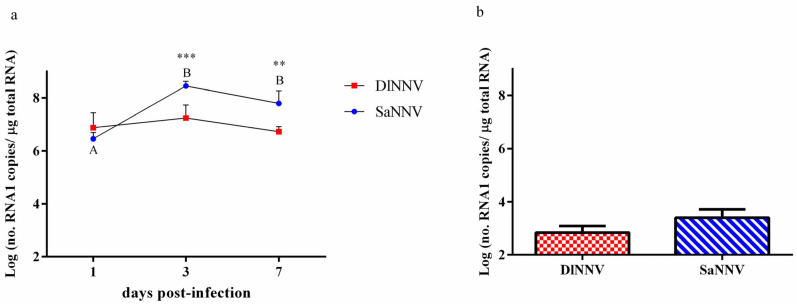
Quantification of the RNA1 segment in brains from juvenile sea bream experimentally challenged with the viral isolates DlNNV (red) or SaNNV (blue). (**a**) Sampled fish; (**b**) surviving fish. Values are mean ± standard deviation (SD) (n = 6). Different letters indicate significant differences within each experimental group (*p* < 0.0001). Asterisks indicate significant differences between viruses at the same sampling time (** *p* < 0.01; *** *p* < 0.001).

Typical signs of disease and mortality were not recorded in any experimental group for 30 days; however, the quantification of the RNA1 segment in brains from randomly sampled sea bream showed differences between DlNNV and SaNNV replication (Figure 1a). In particular, the DlNNV copy number remained constant in fish brain from 1 to 7 dpi, whereas a significant increase in the number of SaNNV RNA1 copies was recorded from 1 dpi (3.3 × 10^6^ copies/μg RNA) to 3 dpi (2.5 × 10^8^) (*p* < 0.0001). In addition, the number of RNA1 copies was significantly higher (*p* < 0.001) in SaNNV-challenged fish than in brains from sea bream inoculated with DlNNV at 3 and 7 dpi (Figure 1a).

The number of RNA1 copies/μg RNA in surviving fish was 7 × 10^2^ and 2.5 × 10^3^ for DlNNV-challenged and SaNNV-challenged fish, respectively (Figure 1b).

### 3.2. Kinetics of Immune Gene Transcription

**Figure 2:**
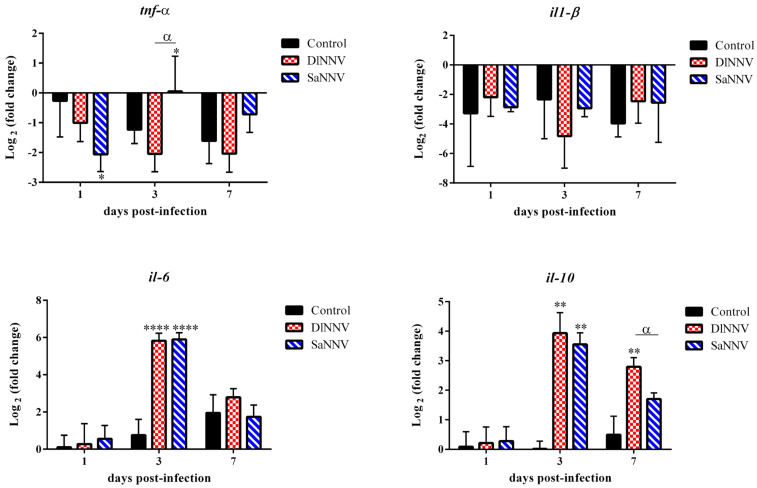
Transcription of genes involved in the inflammatory response in brains from DlNNV- (red) and SaNNV-challenged (blue) juvenile sea bream. Values are mean ± SD (n = 6). Asterisks denote significant differences between control and NNV-challenged fish within each sampling time (* *p* < 0.05; ** *p* < 0.01; **** *p* < 0.0001). α indicates significant differences between DlNNV- and SaNNV-challenged fish (*p* < 0.05).

**Figure 3:**
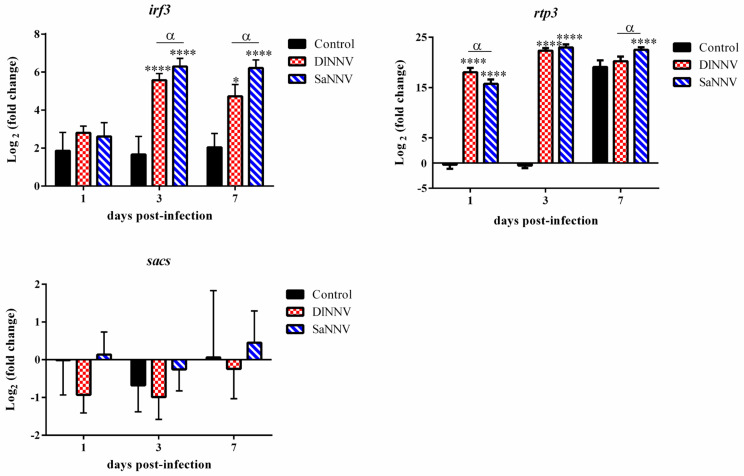
Transcription of genes involved in the IFN-I response in brains from DlNNV- (red) and SaNNV-challenged (blue) juvenile sea bream. Values are mean ± SD (n = 6). Asterisks denote significant differences between control and NNV-challenged fish within each sampling time (* *p* < 0.05; **** *p* < 0.0001). α indicates significant differences between DlNNV- and SaNNV-challenged fish (*p* < 0.05).

**Figure 4:**
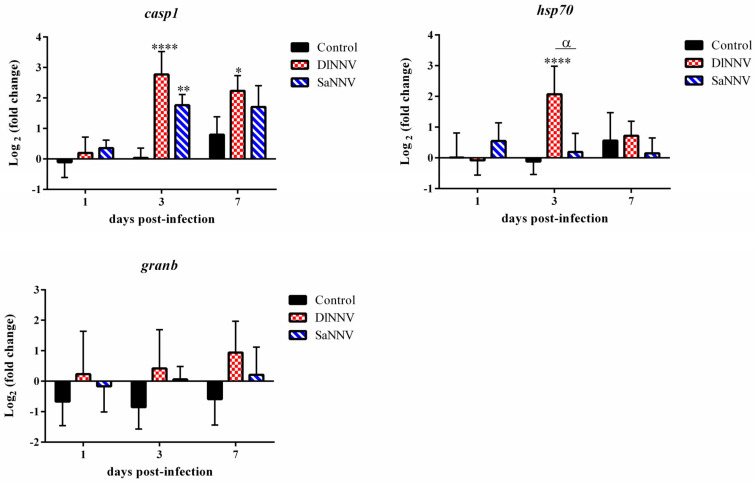
Transcription of the apoptotic genes *casp1* and *granb* and the stress-related gene *hsp70* in brains from DlNNV- (red) and SaNNV-challenged (blue) juvenile sea bream. Values are mean ± SD (n = 6). Asterisks denote significant differences between control and NNV-challenged fish within each sampling time (* *p* < 0.05; ** *p* < 0.01; **** *p* < 0.0001). α indicates significant differences between DlNNV- and SaNNV-challenged fish (*p* < 0.05).

Transcription profiles of a selected set of immune genes have been analysed after infection with both viral isolates at different time points. Overall, a poor response against both NNV isolates was recorded at 1 dpi (only 2 out of 10 analysed genes were significantly modulated by viral infection at that sampling time), whereas the maximum number of genes significantly deregulated by the viral infection was found at 3 dpi (6 genes in each experimental group) (Figure 2, Figure 3 and Figure 4). Most genes were up-regulated after infection; the only significantly (*p* < 0.05) down-regulated gene was *tnf-a* in samples from SaNNV-inoculated fish (at 1 dpi) (Figure 2, Figure 3 and Figure 4). Three genes were not deregulated after infection with any of the viruses tested: *sacs*, *il-1β* and *granb* (Figure 2, Figure 3 and Figure 4).

Specifically, the transcription analysis of genes involved in the inflammatory response (Figure 2) showed that SaNNV infection significantly down-regulated the transcription of the pro-inflammatory gene *tnf-α* at 1 dpi (0.24 FC values), whereas a significant, although low, up-regulation was recorded at 3 dpi (1.04 mean FC values). The transcription of this gene in sea bream injected with DlNNV was statistically similar to that recorded in control fish at all sampling times analysed (Figure 2). On the contrary, the transcription of *il-6* (pro-inflammatory gene) and *il-10* (anti-inflammatory gene) was significantly up-regulated in response to infection with any of the viruses. Particularly, *il-6* transcription was only induced at 3 dpi, and the level of transcription was similar (*p* < 0.0001) in samples from both experimental groups (56.65 and 59.45 mean FC values for DlNNV- and SaNNV-injected sea bream, respectively) (Figure 2). Similarly, both viruses induced *il-10* transcription at 3 dpi (15.28 and 11.76 mean FC values for DlNNV- and SaNNV-injected sea bream, respectively), although the transcriptional induction triggered by the sea bass isolate (DlNNV) lasted longer, since significant transcription was recorded at 7 dpi in fish challenged with this virus (6.93 mean FC values) (Figure 2). Another pro-inflammatory gene analysed, *il-1β*, was not significantly modulated after inoculation with any of the viruses tested (Figure 2).

Regarding the IFN-I system-related genes (Figure 3), *irf3* and *rtp3* transcription was up-regulated following the infection with both viruses, although differences regarding the intensity and/or the temporal profile of the induction were observed when the results from both experimental groups were compared. Particularly, *irf3* FC values were 47.59 and 26.43 at 3 and 7 days after infection with DlNNV, respectively; these values were significantly lower (*p* < 0.05) than those reported in fish injected with the reassortant isolate (78.45 and 74.40 mean FC values at 3 and 7 dpi, respectively) (Figure 3). The *rtp3* gene was highly transcribed in brains from fish infected with both viruses. Thus, early *rtp3* transcription (at 1 dpi) was significantly higher in brains from DlNNV-injected fish; in this experimental group, maximum values were recorded at 3 dpi. At 7 dpi, only samples from SaNNV-infected fish yielded significant *rtp3* induction (Figure 3).

Regarding the apoptotic response (Figure 4), an evident up-regulation of *casp1* transcription was observed at 3 dpi in brains from fish inoculated with any of the viruses (although with similar levels of induction), whereas *granb* was not deregulated (compared to control fish) after viral infections (Figure 4). Finally, *hsp70* transcription was transitorily induced only in brains from sea bream inoculated with the sea bass isolate (Figure 4). The maximum FC value for this gene in DlNNV-infected fish was recorded at 3 dpi (4.88), dropping to basal level at 7 dpi (Figure 4).

### 3.3. Principal Component Analysis

The relationship between the level of transcription of the above-mentioned genes over time and the viral isolate was statistically analysed by principal component analysis (PCA). First of all, in order to identify those genes that are not well represented by the extracted principal components, a study on communalities was performed (Table S1). Genes with communality values lower or equal to 0.4 (*il-1β*, *sacs* and *granb*) were eliminated for subsequent analyses.

**Figure 5:**
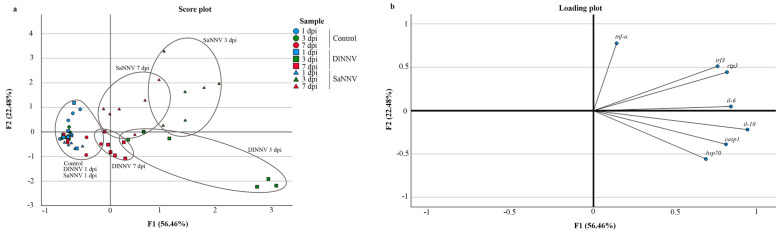
Principal component analysis (PCA). Score plot (**a**) and loading plot (**b**) of the first two principal components (F1 and F2) of a dataset comprised by log_2_ fold change values of immune-related genes in brains from DlNNV- and SaNNV-challenged sea bream.

Two principal components (F1 and F2), explaining 78.94% of the total data variability, were extracted (Figure 5). The score plot (Figure 5a, Figure S1) clustered samples according to the viral isolate and the sampling time from 1 dpi onwards. Thus, samples collected at 3 and 7 dpi from DlNNV-infected sea bream scored negative on F2, whereas samples collected at those times from SaNNV-challenged fish scored positive. Taking these results together, the information depicted in the score plot (Figure 5a) and the loading plot (Figure 5b) shows that higher transcription levels of *hsp70*, *casp1* and *il-10* are associated with DlNNV inoculation, since the arrows point in the direction of that cluster, whereas *tnf-a*, *irf3* and *rtp3* transcription is mainly associated with SaNNV infection in sea bream.

Regarding correlation analyses, F1 is positively correlated (r ≥ 0.7, *p* < 0.05) with *il-10*, *il-6*, *rtp3*, *casp1*, *irf3* and *hsp70* transcription, whereas F2 is positively correlated with the FC values calculated for *tnf-α* (Figure 5b, Table S2). In addition, this analysis evidenced a positive correlation between genes (Table S3). The tightest significant correlations (r ≥ 0.7, *p* < 0.05) were stablished between the following pairs of genes: *irf3* and *rtp3*; *il-10* and *il-6*; *il-10* and *casp1*; *hsp70* and *casp1*; *hsp70* and *il-10*.

## 4. Discussion

Sea bream is highly resistant to viral infections, acting as an asymptomatic carrier of several fish viruses, including RGNNV [18,25,26]. In fact, this fish species has been classically reported to be susceptible only to the lymphocystis disease, caused by the lymphocystis disease virus [27]; however, recent studies have evidenced high mortalities in sea bream hatcheries caused by RGNNV/SJNNV betanodaviruses [5,10,13]. In fact, infections caused by these viral isolates are of major concern for the Mediterranean aquaculture industry nowadays [1].

The outcomes of viral infections depend on specific virus–host interactions. Understanding the mechanisms responsible for these interactions is crucial to control viral diseases in aquaculture. For this reason, in this study, we have analysed the transcription of a selected set of immune genes in brains from sea bream inoculated with a betanodavirus isolated from this fish species (SaNNV, RGNNV/SJNNV) in comparison with the response elicited by DlNNV (RGNNV), which was isolated from sea bass, in an attempt to shed some light on the reasons underlying sea bream resistance to RGNNV infections. Both isolates were obtained from specimens showing evident signs of disease.

As a first step, in order to establish the virulence of both isolates to sea bream, two groups of fish were challenged with each virus and monitored for 30 days. After that time, no mortalities or symptoms were recorded in any experimental group, which is coherent with the low number of viral genome copy numbers recorded in fish surviving both viral infections (Figure 1b). The absence of mortality after sea bream inoculation with an RGNNV isolate agrees with previous studies [18,28,29], and the resistance of sea bream to the disease caused by RGNNV is also supported by field observations. Thus, Cherif et al. [30] detected asymptomatic PCR-positive sea bream reared next to diseased sea bass in a Tunisian farm. Regarding SaNNV, our results are not in concordance with several studies reporting RGNNV/SJNNV-associated mortality in juvenile sea bream [5,10,13]. However, these previous reports, although demonstrating sea bream susceptibility to RGNNV/SJNNV, refer to natural outbreaks in fish farms. To the best of our knowledge, only Vazquez-Salgado et al. [31] have described sea bream mortality after experimental infection with RGNNV/SJNNV isolates (obtained from Senegalese sole and sea bream) at post-larval stages, using 1-g specimens. Most of the sea bream challenges reported in literature have been performed with larvae [32,33], which suggests that size may be a relevant factor regarding sea bream susceptibility to RGNNV/SJNNV isolates under experimental conditions and may explain the lack of mortality in the current study, which was performed with 3-g sea bream. This hypothesis is supported by previous observations derived from natural outbreaks, describing high mortalities only in larvae under ca. 35 days post-hatching [10,13,17].

The absolute quantification of the viral genome has demonstrated that only SaNNV replicated in sea bream brains, with a significant increase in the RNA1 copy number from 1 to 3 dpi (Figure 1a). To our knowledge, this is the first report of RGNNV/SJNNV replication in brains from juvenile sea bream specimens; previous analyses refer to RGNNV/SJNNV replication in sea bream larvae [32,33]. The high number of SaNNV genome copies in fish sampled at 3 dpi (2.5 × 10^8^ RNA1 copies/μg RNA), higher than that reported in surviving RGNNV-challenged sea bass [22], is also noteworthy, supporting the great resistance of sea bream to viral infections, which makes especially interesting to look into the antiviral response of this fish species. Regarding RGNNV, unlike the results recorded in this work, previous studies have described replication in sea bream brain [28,29,34,35]. The results obtained in the present study may indicate that the isolate DlNNV does not replicate in sea bream, revealing differences associated with the specific viral strain used, or that viral replication takes place earlier, before 1 dpi. In order to further investigate how DlNNV and SaNNV interact with the sea bream immune system in juvenile specimens, the transcription of some immune genes was quantified and compared.

Transcriptional deregulation upon NNV infection in sea bream is not as evident as it has been described in other susceptible fish species, such as sea bass, Senegalese sole, zebrafish (*Danio rerio*) or Atlantic halibut (*Hippoglossus hippoglossus*), especially regarding the inflammatory response [36,37,38]. The “mild” inflammatory response detected in the present work has also been described in previous studies after sea bream inoculation with RGNNV, RGNNV/SJNNV and SJNNV/RGNNV [32,34] and can be related to the lack of typical symptoms of disease in this fish species, even when mortalities are recorded [12,39]. In fact, *il-1β*, which has not been induced by the viral infections in this study, has been proven to cause neurodegeneration in grouper brain [40], and *tnf-α*, which has been down-regulated by SaNNV infection (Figure 2), although it has been suggested to protect neurons in NNV-infected grouper cells [40], has also been proven to induce neuropathology associated with viral infections in mammals [41,42]. Additionally, the long-lasting induction of the anti-inflammatory gene *il-10* in DlNNV-challenged fish (Figure 2) can be considered as an additional strategy to control inflammation, as it has been previously suggested by comparing *il-10* induction in RGNNV-infected sea bass (susceptible to RGNNV) and sea bream (resistant) [35].

In the present study, SaNNV-triggered response is characterized by the highest FC values for the IFN-I-related genes (*irf3* and *rtp3*) at 3 and 7 dpi (Figure 3). Similarly, in other susceptible fish species, such as sea bass or Senegalese sole, the immune response against NNV infections is dominated by a noticeable induction of IFN-I system-related genes [37,43,44,45,46]. These results suggest that the IFN-I-system response may also be key to determine sea bream resistance or susceptibility to NNV. Furthermore, Garcia-Alvarez et al. [32] also reported significant transcription of IFN-I system-related genes and a similar kinetics of immunogene transcription in RGNNV/SJNNV-challenged sea bream larvae, which may indicate that the innate immune response against RGNNV/SJNNV is similar in larval and juvenile specimens.

In addition, PCA has demonstrated a strong correlation between *irf3* and *rtp3* transcription (r = 0.875, *p* < 0.001). In this regard, a recent study has demonstrated *rtp3* induction in sea bass following poly I:C inoculation, indicating that *rtp3* can be considered as an IFN-stimulated gene (ISG) [47].

Moreover, *rtp3* is the only analysed gene that was up-regulated by infection at 1 dpi. At that time, unlike it was observed at 3 and 7 dpi, *rtp3* induction was significantly higher in brains from DlNNV-challenged sea bream than in samples from SaNNV-inoculated fish, suggesting that this higher early *rtp3* induction could be behind the inhibition of DlNNV replication recorded in this study (Figure 1a). However, knowledge on the antiviral activity of this gene is scarce and limited to Asian sea bass (*Lates calcarifer*), where an important role in fish resistance to NNV infections has been suggested [48]. Concerning *sacs*, previous studies have suggested a putative role for this gene in controlling NNV infections on the basis of the high level of transcription recorded in several fish species, such as Senegalese sole, sea bass and sea bream [33,36,43,49]. Despite this, knowledge on the activity of this gene is scarce and limited to reovirus-infected grass carp (*Ctenopharyngodon idella*), where an anti-apoptotic effect has been suggested [50]. This is the first study describing lack of *sacs* modulation by NNV infection in sea bream, since the induction of this gene has been previously reported in sea bream after RGNNV inoculation [34].

IRF3 is a transcription factor activated upon viral infection, leading the transcription of *ifn-I* genes. In addition to this role, recent studies have demonstrated that IRF3 can also induce apoptosis through its interaction with the Bax protein and its subsequent mitochondrial translocation [51,52,53]. The apoptotic role of IRF3 has been demonstrated during infections with several RNA viruses, such as paramyxovirus, vesicular stomatitis virus (VSV) and encephalomyocarditis virus (EMCV) [53,54]. Although apoptosis can be a mechanism to suppress viral replication, uncontrolled apoptosis derived from an exacerbated IFN-I response could contribute to tissue lesions. Whether the apoptotic role of IRF3 is involved in increasing or controlling the extent of the disease in sea bream remains to be investigated.

Other apoptotic genes analysed in this study have been *granb* and *casp1*, although only *casp1* was significantly modulated by viral infection (Figure 4). Granzymes are serine proteases within the secretory granules of T lymphocytes and natural killers, causing cell death by necrotic and apoptotic pathways [55]. The lack of *granb* induction after NNV infections recorded in this study could indicate a minor role of the cell-mediated cytotoxic (CMC) response in the clearance of both viruses, as it has been previously suggested for an RGNNV/SJNNV isolate in sea bream larvae [32], or it may be a consequence of the minor role of this granzyme, compared with granzyme A, within the CMC response against NNV infection in sea bream, as it has been previously demonstrated [56]. In order to get more insight into the role of sea bream CMC in controlling SaNNV and/or DlNNV infections, studies regarding the transcription of other genes related with the CMC should be conducted.

Caspase1 is a protease that causes cell death by pyroptosis [57] and can also recruit immune cells to the infection site. The results derived from the present study (Figure 4) suggest a more relevant role of sea bream *casp1* in the clearance of NNV isolated from sea bass (DlNNV) than in limiting the infection of the sea bream isolate (SaNNV). Furthermore, PCA has also suggested a relation between *casp1* and *hsp70* transcription (r = 0.723, *p* < 0.01) (Table S3), which is reinforced by a study demonstrating that *hsp70* has an important function in apoptosis regulation in mammals [58]. The induction of *hsp70* was only observed in DlNNV-inoculated sea bream, and further studies are required in order to clarify the role of this gene in the course of RGNNV infection in sea bream, since HSP70 has been reported to have both, antiviral and pro-viral effects. Thus, this protein has been demonstrated to be a host factor promoting RGNNV replication in grouper (*Epinephelus coioides*) and medaka (*Oryzias latipes*) cells [59,60], in addition to being involved in both, adaptative and innate immune responses [61].

PCA clustered samples according to the viral isolate used at 3 and 7 dpi, revealing the coherence of the results, whereas samples collected at 1 dpi from challenged fish clustered together with control samples (Figure 5a). This analysis also evidenced differences in the immune response against DlNNV and SaNNV, even though none of the isolates caused mortality or symptomatology. These differences were not observed when the immune response elicited in sea bream larvae by RGNNV/SJNNV and SJNNV/RGNNV was compared [32], and they can be the key to understanding the mechanism behind the inhibition of RGNNV (DlNNV isolate) replication in sea bream.

In summary, this study has shown that the innate immune response against NNV in sea bream resembles that described in other susceptible fish species regarding the high induction of IFN-I system-related genes; however, the inflammatory and apoptotic responses are reduced. A closer look into the transcriptional response has evidenced some differences between the response elicited by the isolates obtained from sea bass (DlNNV, which is unable to replicate in sea bream) and sea bream (SaNNV, which replicates in sea bream). Thus, the response against DlNNV is characterized by significantly higher *rtp3* transcription early after the infection, longer-lasting transcription of the anti-inflammatory gene *il-10* and stronger induction of both *casp1* and *hsp70*. Therefore, these genes should be targets for future studies in order to elucidate their role in hampering the replication of the sea bass isolate in sea bream. In addition, our results suggest that the balance between viral replication and host immune defence results in the control of the VNN disease in 3-g sea bream inoculated with an RGNNV/SJNNV isolate. The challenge is to understand the mechanisms underlying this balance, which could contribute to developing effective prophylactic measures for sea bream larvae, for which VNN is a serious threat.

## Data Availability

Dataset available on request from the authors.

## Supplementary Materials

The following supporting information can be downloaded at: https://www.mdpi.com/article/10.3390/pathogens13060478/s1, Figure S1: Principal component analysis (PCA). Score plot of the first two principal components (F1 and F2) of a dataset comprised by mean log_2_ fold change values of immune-related genes in brain from DlNNV- and SaNNV-challenged sea bream; Table S1: Principal component analysis data. Communality values ≤ 0.4 are shown in bold; Table S2: Principal component analysis data. Correlation between the principal components extracted (F1 and F2) and the mean log_2_ fold change values for each gene. Significant correlations (r ≥ 0.7; *p* < 0.05) are shown in bold. * *p* < 0.05; Table S3: Principal component analysis data. Correlation between the mean log_2_ fold change values for each gene. Significant correlations (r ≥ 0.7; *p* < 0.05) are shown in bold. * *p* < 0.05.
