# Supplementary material for: Comparative Analysis of Immune Gene Transcription in Sea Bream (Sparus aurata) Challenged with RGNNV or RGNNV/SJNNV Betanodaviruses
Source: Pathogens. 2024 Jun 4;13(6):478. doi: 10.3390/pathogens13060478 (PMC11207047; doi:10.3390/pathogens13060478)
Supplement: Supplementary file 1 [file pathogens-13-00478-s001.zip › pathogens-2949546-supplementary.pdf]

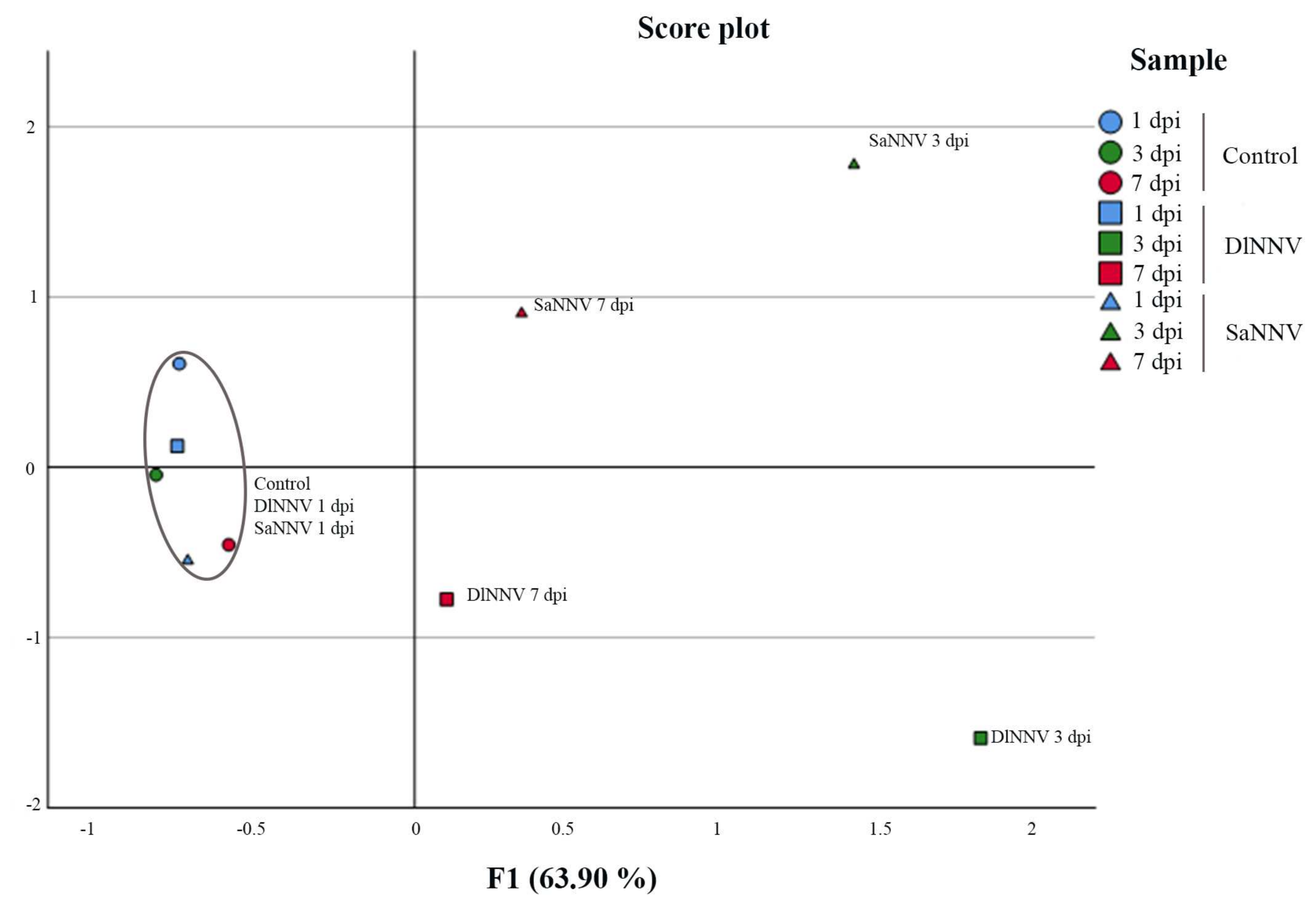

Score plot
Sample
1 dpi
3 dpi
7 dpi
Control
1 dpi
3 dpi
7 dpi
DlNNV
1 dpi
3 dpi
7 dpi
SaNNV
SaNNV 3 dpi
SaNNV 7 dpi
Control
DlNNV 1 dpi
SaNNV 1 dpi
DlNNV 7 dpi
DlNNV 3 dpi
2
1
0
-1
-2
-1
-0.5
0
0.5
1
1.5
2
F1 (63.90 %)

**Communalities**	
tnf-α	0.525
rtp3	0.851
il-1β	**0.084**
irf3	0.859
sacs	**0.275**
casp1	0.753
il-6	0.670
il-10	0.913
hsp70	0.765
granb	**0.365**

Correlation							
	tnf-α	*il-6*	*il-10*	*irf3*	*rtp3*	*casp1*	*hsp70*
F1	0.140	**0.837***	**0.938***	**0.756***	**0.813***	**0.807***	**0.684***
F2	**0.777***	0.048	-0.218	0.512*	0.445*	-0.388	-0.557*

**Correlation**						
	tnf-α	*il-6*	*il-10*	*irf3*	*rtp3*	*casp1*
il-6	0.121					
il-10	0.003	**0.790***				
irf3	0.367	0.539	0.551			
rtp3	0.285	0.68	0.642	**0.875***		
casp1	-0.132	0.522	**0.808***	0.452	0.455	
hsp70	-0.133	0.485	**0.756***	0.198	0.235	**0.723***
